# Residue‐level mapping of crowding effects on protein phase separation

**DOI:** 10.1002/pro.70546

**Published:** 2026-03-25

**Authors:** Wei Chen, Jacob M. Shaffer, Christine D. Keating, Scott A. Showalter

**Affiliations:** ^1^ Department of Chemistry The Pennsylvania State University University Park Pennsylvania USA; ^2^ Department of Biochemistry and Molecular Biology The Pennsylvania State University University Park Pennsylvania USA

**Keywords:** condensate, crowding, intrinsically disordered protein, NMR, phase separation

## Abstract

Protein liquid–liquid phase separation has emerged as a key mechanism in cellular organization. While the crowded environment inside cells is expected to influence this process, how crowding shapes the chemical environment and impacts protein phase separation remains largely unknown. Here, we use NMR spectroscopy to map residue‐level crowding effects on the intrinsically disordered region of RNA polymerase II under different conditions, including polymer‐ and protein‐based crowders, as well as reconstituted *E. coli* cytosol. We find a general trend of enhanced protein self‐interactions across all conditions, but also distinct chemical environments that depend on crowder identity, reflecting changes in preferential interactions. Given the widespread use of polymer crowders, our results provide a strategy to evaluate their chemical influence and to design more physiologically relevant in vitro crowding models. More broadly, this framework enables systematic probing of residue‐level influences in complex, cell‐like environments.

Biomolecular phase separation has emerged as a leading explanation for cellular compartmentalization through the segregation of macromolecules into liquid‐like condensates (Banani et al. 2017). Phase separation is driven by multivalent intermolecular interactions that are often mediated by intrinsically disordered regions (IDRs) of proteins (Chen et al. 2024). In vitro studies of phase separation typically focus on purified proteins, and polymers such as polyethylene glycol (PEG), Ficoll, and dextran are frequently added to enhance phase separation. While sequence and compositional determinants of protein phase separation have been extensively studied (Vendruscolo and Fuxreiter 2023), how crowding influences the phase behavior of proteins is less well understood. Crowding agents are commonly assumed to be inert and simply occupy space to mimic macromolecular crowding in the cell. However, extensive studies have shown that polymer crowders and biological macromolecules can have contrasting effects on processes such as protein folding, denaturation, and enzyme activity. In the context of biomolecular phase separation, it has been shown that polymer crowders can engage in preferential interactions (Annunziata et al. 2002; Lee and Lee 1981; Qian et al. 2022; Stringer et al. 2023; Wang et al. 2012; Wang and Annunziata 2007) and even co‐condense with a variety of biomolecules (André et al. 2023; Marianelli et al. 2018; Qian et al. 2022). In addition, a large number of reported phase‐separating proteins lack data in the absence of crowding agents (Andre and Spruijt 2020). Despite their widespread use, the ability of crowders to recapitulate the effects of cellular crowding on protein phase separation, specifically how they shape the chemical environment around proteins, remains largely unexplored.

To address this gap, we investigated the phase separation behavior and the chemical environments sensed by the C‐terminal domain (CTD) of the RNA polymerase II (Pol II) across different crowding conditions, formed by polymer‐ and protein‐based crowding agents, as well as reconstituted *E. coli* cytosol. The intrinsically disordered CTD has been attributed a significant role in the formation of dynamic Pol II clusters in cells via phase separation (Boehning et al. 2018). It consists of tandem repeats of the amino acid sequence YSPTSPS (Figure 1a), whose repetitive and low‐complexity nature enables multivalent interactions that could support phase separation, while also making the CTD a simple model for probing crowder influences. Phase separation of the CTD has been demonstrated for both the yeast CTD (26 repeats) and human CTD (52 repeats) (Boehning et al. 2018; Flores‐Solis et al. 2023; Linhartova et al. 2024; Zhang et al. 2024), with the number of repeats correlating with phase separation tendency in vitro and in vivo. Polymer crowders such as dextran have been widely used to promote CTD phase separation in vitro (Boehning et al. 2018; Flores‐Solis et al. 2023; Linhartova et al. 2024; Zhang et al. 2024). To dissect how crowding mediates CTD phase separation, we systematically compared the effects of three categories of crowders—polymers (PEG, Ficoll, and dextran), proteins (bovine serum albumin (BSA) and lysozyme), and reconstituted *E. coli* cytosol (Sarkar et al. 2013; Wang et al. 2010), and mapped the microscopic environment they create.

**FIGURE 1:**
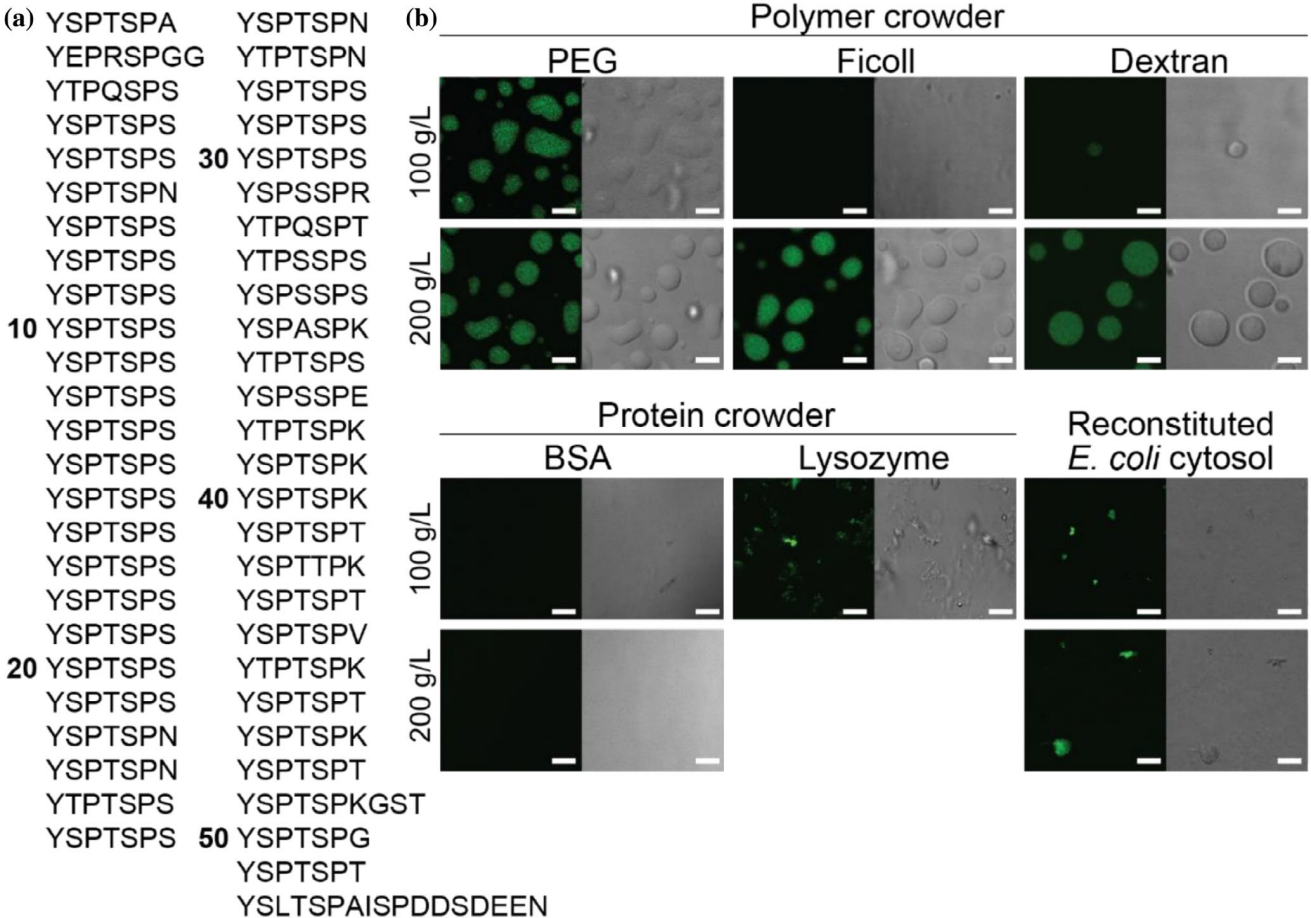
Crowder identity determines the phase separation behaviors of the human CTD (hCTD). (a) The sequence of hCTD contains 52 tandem heptad repeats with the consensus sequence of YSPTSPS. (b) Confocal fluorescence and differential interference contrast (DIC) imaging show that phase separation of 25 μM MBP‐hCTD, fluorescently labeled with Alexa Fluor 488, depends on the identity and concentration of crowders. While polymers promoted CTD droplet formation, proteins and reconstituted *E. coli* cytosol did not. Lysozyme and *E. coli* cytosol led to the formation of irregular CTD aggregates. Scale bar: 5 μm. Experiments were carried out in 50 mM HEPES pH 7.5 and 150 mM NaCl. Fluorescence images were false colored with adjusted brightness/contrast for clarity.

We first compared the phase behavior of the human CTD (hCTD) across different crowding conditions. Among three commonly used polymer crowders, PEG (MW 8 kD) is linear, dextran (MW 9–11 kD) is a slightly branched (~5%), and Ficoll (MW 70 kD) is highly branched. The CTD phase separated into liquid droplets in the presence of all three polymers at a crowder concentration of 200 g/L (Figure 1b). PEG was the most effective driver, producing abundant droplets even at 100 g/L, whereas Ficoll had no effect and dextran yielded only rare small droplets. In contrast to polymers, globular proteins are more rigid and can provide nonspecific protein–protein interactions that more closely resemble those in cells. We therefore examined two protein‐based crowders—BSA (MW 66 kD, pI 5) and lysozyme (MW 14 kD, pI 9), which are oppositely charged at pH 7.5. Under these conditions, BSA did not promote CTD phase separation, while lysozyme induced irregular aggregate formation. To more closely mimic cellular composition, we reconstituted *E. coli* cytosol at 100 and 200 g dry weight/L from lyophilized lysate (Sarkar et al. 2013; Wang et al. 2010), reflecting physiological macromolecular concentrations (100–400 g/L) (Luby‐Phelps 2000). The reconstituted cytosol contained soluble cytoplasmic proteins, nucleic acids, and ions, but excluded cell walls, membrane fragments, and insoluble proteins. Similar to lysozyme, reconstituted cytosol led to CTD aggregation, though on a smaller scale with fewer aggregates. Taken together, these results show that even at the same macromolecular concentration, the identity of the crowder determines CTD phase behavior. Notably, only polymer crowders supported liquid condensate formation under our conditions.

To gain insights into how macromolecular crowding modulates CTD interactions relevant to phase separation, we used NMR spectroscopy to map the chemical environments of the CTD at residue‐level resolution under the same conditions (50 mM HEPES pH 7.5, 150 mM NaCl, and 100 g/L crowder). NMR has been widely applied to study how crowding influences protein stability (Wang et al. 2012), folding (Tokuriki et al. 2004), and association (Guseman and Pielak 2017), but its use to elucidate residue‐level crowding effects in the context of phase separation remains largely unexplored. Here, we focused on a shorter CTD construct containing 12 YSPTSPS repeats (CTD12), chosen for its high expression yield required for NMR. Our working assumption is that the dominant interactions underlying both hCTD and yCTD phase separation in the presence of polymer crowders (Boehning et al. 2018; Flores‐Solis et al. 2023; Linhartova et al. 2024; Zhang et al. 2024) are encoded within the repeating YSPTSPS heptads and can therefore be probed using a minimal CTD12 construct. This assumption is supported by a previous NMR study showing that residue‐level interactions in yCTD are recapitulated in a 3‐repeat peptide (Flores‐Solis et al. 2023). While our simplified CTD12 construct does not capture potential emergent interactions arising from repeat numbers beyond 12, sequence features unique to the human CTD, or contributions from tags, it enables identification of residue‐level interaction features within the YSPTSPS repeats that are modulated by crowding.

The CTD is rich in prolines (~30%), which are prevalent in other IDRs (Uversky 2013) and have been implicated in phase separation through contacts with tyrosines and *cis‐trans* isomerization (Flores‐Solis et al. 2023; Linhartova et al. 2024). However, prolines are traditionally challenging for protein NMR because they lack amide hydrogens and thus are invisible in ^1^H‐^15^N‐HSQC spectra (Figure 2a). To overcome this limitation, we carried out ^13^C direct‐detect ^13^C, ^15^N‐CON experiments (Figure 2b), which allowed a direct visualization of prolines with improved spectral dispersion. We observed both P_3_ and P_6_ in *trans* conformation and also *cis*‐P_3_ (10% of P_3_ population). We focused on the internal repeats of CTD12, rather than the terminal ones, as they better represent the full‐length CTD. In both ^1^H‐^15^N‐HSQC and ^13^C, ^15^N‐CON spectra, residues with the same numbering in these Y_1_S_2_P_3_T_4_S_5_P_6_S_7_ repeats (excluding termini) clustered as a single peak, reflecting their averaged chemical environment (Figure 2).

**FIGURE 2:**
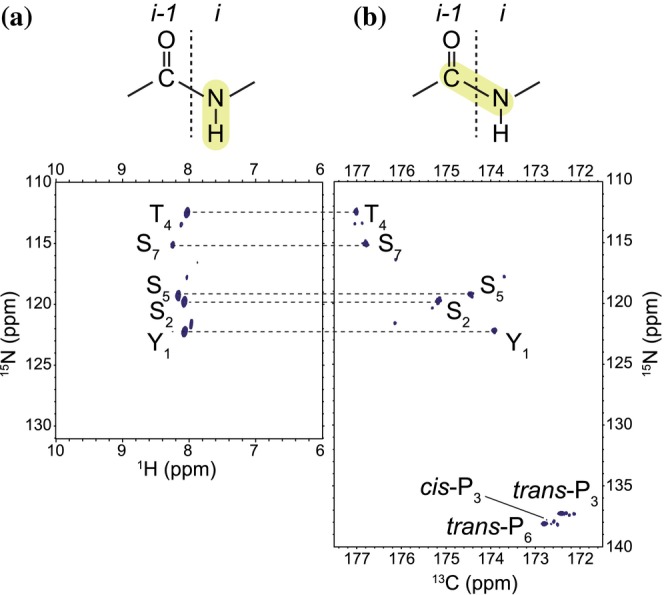
Carbon direct‐detect NMR provides enhanced resolution and complete residue coverage for the CTD. (a) Proton‐detected ^1^H, ^15^N‐HSQC spectrum of CTD12 shows narrow chemical shift dispersion in the ^1^H dimension and lacks signals from proline residues, limiting residue‐level analysis. (b) Carbon‐detected ^13^C, ^15^N‐CON spectrum exhibits wider dispersion in the ^13^C dimension and direct visualization of proline residues, allowing comprehensive mapping of the CTD. Experiments were carried out using 425 μM CTD12 in 50 mM HEPES pH 7.5, 150 mM NaCl, 1 mM DSS, and 10% D_2_O.

We first performed ^13^C, ^15^N‐CON experiments for CTD12 at two concentrations (425 μM and 1 mM) (Figure S1a, Supporting Information) without crowders and analyzed weighted‐average chemical shift perturbations (CSPs) across the ^13^C and ^15^N dimensions. At 1 mM, Y_1_, S_2_, and S_5_ showed the largest concentration‐dependent CSPs (Figures 3a and 4a), reflecting CTD12 self‐interactions enhanced by higher concentration and consistent with previous studies implicating tyrosines in CTD phase separation (Flores‐Solis et al. 2023; Linhartova et al. 2024) as well as in the stickers‐and‐spacers model (Martin et al. 2020). We then examined CSPs for 425 μM CTD12 in the presence of 100 g/L PEG, dextran, BSA, lysozyme, and reconstituted *E. coli* cytosol, under the same crowder and buffer conditions used in the confocal assays for hCTD. Although these NMR samples did not appear turbid, the interactions underlying phase separation can still be present in the dilute phase, as previously reported in other NMR studies (Flores‐Solis et al. 2023; Martin et al. 2020). The volume fraction of 100 g/L PEG, BSA, and lysozyme were calculated to be 8.93%, 7.09%, and 6.85%, respectively, using reported or calculated densities (not available for the other crowders) (Fischer et al. 2004; Soranno et al. 2014). Because crowders are NMR silent, the observed CSPs directly report the chemical environments sensed by CTD12. Different crowders produced distinct residue‐level CSP magnitudes and patterns (Figures 3a and S1), with PEG inducing the largest CSPs (Figure 3b,c), consistent with its strong ability to drive CTD phase separation. Notably, *cis*‐P_3_ (for which only ^15^N CSPs were available) displayed the largest CSP among all residues despite its small population (10% for 425 μM, 9% for 1 mM, 8% for BSA, 4% for PEG, and undetectable for lysozyme and reconstituted *E. coli* cytosol) (Figure 3c,d). This indicates that *cis*‐P_3_ is highly sensitive to concentration and environmental changes, consistent with previous findings that proline‐tyrosine contacts and proline *cis‐trans* isomerization are important for CTD phase separation (Flores‐Solis et al. 2023; Linhartova et al. 2024). Overall, these observations indicate that different crowders create distinct microscopic chemical environments around the CTD.

**FIGURE 3:**
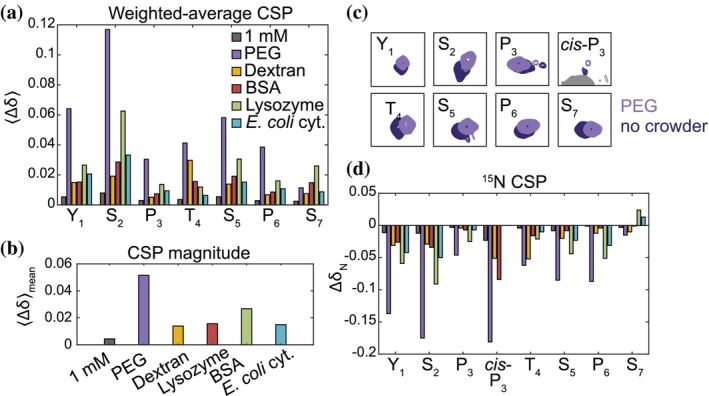
Residue‐level NMR mapping reveals distinct chemical environments induced by different crowders. (a) Weighted average chemical shift perturbations (CSPs), ⟨∆δ⟩, for each residue in the YSPTSPS repeat show that different crowders alter the local chemical environments in unique ways. Increasing CTD12 concentration (1 mM vs. 425 μM) also caused distinct CSPs for different residues, reflecting CTD self‐interactions. (b) Mean CSP magnitude under each condition, reflecting the overall extent of crowding‐induced perturbation. (c) Example of residue‐specific peak shifts in the ^13^C, ^15^N‐CON spectrum upon addition of PEG. (d) ^15^N CSPs highlight pronounced concentration and crowding effects on *cis*‐P3.

**FIGURE 4:**
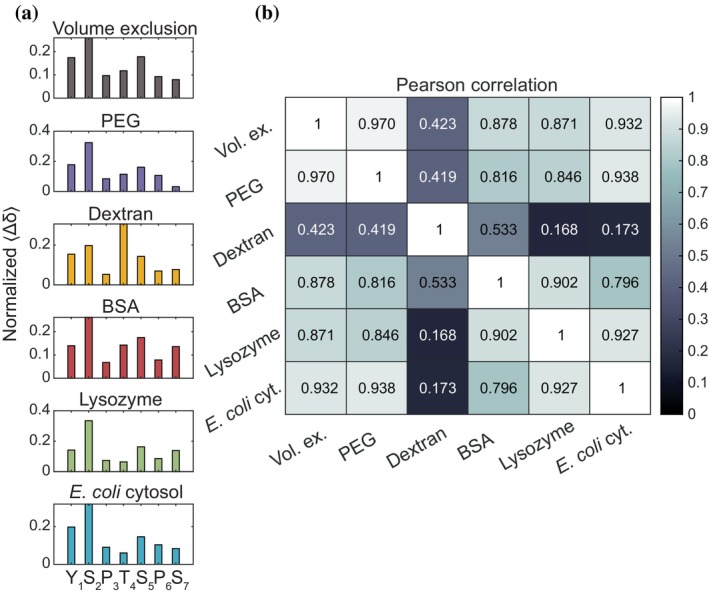
NMR chemical shift perturbation (CSP) analysis compares residue‐level influences across crowding conditions. (a) Normalized CSP profiles of the CTD12 under different crowding conditions. A self‐reference profile was obtained from CSPs at 1 mM CTD12 without crowders. Similarity to this profile suggests enhanced self‐interactions, whereas deviations reflect additional residue‐specific influences from distinct crowder environments. (b) Heatmap of Pearson correlation coefficients calculated from combined ^15^N and ^13^C CSP components between conditions, quantifying similarity among different crowded environments and the CTD12 self‐reference.

To interpret the crowder‐dependent CSPs, we normalized the weighted‐average CSP profile for each condition such that the sum equals one (Figure 4a), and compared the normalized profiles across conditions. We used the CTD12 profile at high concentration (1 mM, corresponding to 8.74 g/L) as a self‐reference that captures residue‐level changes associated with enhanced CTD12 self‐interactions. We then assessed the extent to which CSP patterns under different crowding conditions resembled this self‐reference. The normalized CSP profiles showed that all crowding conditions, except dextran, exhibited the largest CSP at S_2_, followed by Y_1_ or S_5_, similar to the self‐reference (Figure 4a). This similarity is consistent with crowding generally enhancing CTD12 self‐interactions through increased effective concentration, to which volume exclusion is expected to contribute. At the same time, each crowder displayed distinct deviations in its CSP pattern. PEG had less effect at S_7_, lysozyme had more effect at S_7_ and less at T_4_, and reconstituted *E. coli* cytosol had less effect at T_4_, when compared to the self‐reference (Figure 4a). These deviations suggest additional contributions such as changes in preferential interactions, either associative or repulsive, between CTD12, the crowder, and buffer components, as well as potential modulation of how CTD12 self‐interacts under different crowded environments.

To quantify the similarity between conditions, we calculated Pearson correlation coefficients (*r*) across combined ^15^N and ^13^C CSP components (Figure 4b). This analysis captures similarities in the direction and relative residue‐to‐residue magnitude of peak movement in the ^13^C, ^15^N‐CON plane, reflecting changes in the local chemical environment at the residue level. Among all crowders, PEG showed the highest correlation with the CTD12 self‐reference (*r* = 0.937), suggesting that PEG induces a chemical environment that closely resembles CTD12 self‐interactions at higher concentration, consistent with a dominant contribution from increased effective CTD12 concentration. In contrast, dextran exhibited the lowest correlations with other conditions (*r* = 0.570–0.692), consistent with its distinct CSP pattern characterized by the largest perturbation at T_4_ instead of S_2_ (Figure 4a), which suggests that its chemical environment differs substantially and that preferential interactions might play a larger role.

Interestingly, lysozyme and PEG showed higher similarities to reconstituted *E. coli* cytosol (*r* = 0.957 and *r* = 0.933) than BSA did (*r* = 0.688), despite that lysozyme and BSA are both protein‐based crowders. This trend mirrors their respective similarity to the self‐reference, with PEG (*r* = 0.937), lysozyme (*r* = 0.824), and *E. coli* cytosol (*r* = 0.859) all higher than BSA (*r* = 0.602). A possible explanation is that, in the complex cytosolic mixture, weak interactions between CTD12 and many different components average out, making self‐interactions more prominent. This effect is captured by crowders that enhance self‐interactions, such as PEG and lysozyme. For crowders such as BSA and dextran, there may be changes in preferential interactions and self‐interactions leading to more residue‐specific deviations from the self‐reference. We noted that, although PEG, lysozyme and reconstituted *E. coli* cytosol exhibited similar normalized CSP profiles and high Pearson correlation coefficients, PEG had substantially larger CSP magnitudes (Figure 3a,b), indicating distinct degrees of crowding effects consistent with the different hCTD phase behaviors observed under these conditions (droplets with PEG vs. aggregates with lysozyme and reconstituted *E. coli* cytosol). These results motivate a closer examination of the physical mechanisms underlying PEG‐induced crowding effects, particularly given its widespread use as a nominally inert polymer crowder.

Extensive studies have shown that, while excluded volume effects dominate many crowding‐driven protein behaviors (Bhat and Timasheff 1992; Park et al. 2020; Soranno et al. 2014; Zosel et al. 2020), PEG can participate in complex, multifaceted interactions beyond purely steric effects that significantly influence processes including protein phase separation (André et al. 2023; Annunziata et al. 2002; Bloustine et al. 2006; Lee and Lee 1981; Qian et al. 2022; Stringer et al. 2023; Wang and Annunziata 2007). Thermodynamic (Annunziata et al. 2002; Lee and Lee 1981; Qian et al. 2022; Wang and Annunziata 2007), light‐scattering (Bloustine et al. 2006), single‐molecule fluorescence (Stringer et al. 2023), and computational (Chao et al. 2017) studies have shown that PEG can engage in weak, transient interactions with proteins, including favorable interactions with both hydrophobic and hydrophilic groups (Chao et al. 2017; Knowles et al. 2015). We also compared PEG polymer to ethylene glycol (EG) monomer and observed distinct residue‐level CSP profiles (Figure S2), which likely reflect polymer‐specific effects such as excluded volume and difference in hydration (Chu et al. 2023; Knowles et al. 2011; Liebau et al. 2024; Semmeq et al. 2024). In the context of phase separation, recent work further suggests that PEG‐induced condensate formation correlates with the transition of PEG from the dilute to the semidilute regime beyond its critical overlap concentration (C*), where polymer chains begin to overlap and formed entangled network (Bullier‐Marchandin et al. 2025). For PEG of approximately 8 kD, C* has been reported to lie between 5 and 10% (w/v) (Liebau et al. 2024; Soranno et al. 2014). Many in vitro studies, including ours, operate within or above this concentration range, where polymer network can hinder protein diffusion and influence protein–protein interactions (Bullier‐Marchandin et al. 2025).

Collectively, these findings highlight that PEG can influence protein phase separation through a combination of excluded volume effects, weak preferential interactions, and its transition from the dilute to the semidilute regime. This complexity extends to sugar‐based crowders as well (Redvanly et al. 2026). Notably, dextran, the most used crowder in CTD studies, exhibited the largest deviation from both other crowders and the CTD12 self‐reference, indicating distinct crowders can reshape residue‐level interactions in different ways. These observations underscore the need for residue‐level approaches for comparing how specific crowding environments modulate protein behavior.

Taken together, we showed that crowder identity shaped the residue‐level chemical environment experienced by an IDR and had drastic effects on protein phase separation. Because polymers often poorly recapitulate cellular crowding (Tyrrell et al. 2015), more complex mixtures and cell lysates are increasingly used (Davis et al. 2020; Sarkar et al. 2013). Given the common use of polymer crowders in phase separation studies, our results provide a foundation for designing more physiologically relevant in vitro models by allowing direct comparison to cell‐like environments such as nuclear or cytoplasmic extracts, and ultimately to cellular environments via in‐cell NMR (Theillet and Luchinat 2022). More broadly, this work presents a proof of concept for probing residue‐level environmental influence, including those in the cellular milieu where physiological crowding and native interactions are present, to systematically decode the molecular grammar for protein phase separation in crowded environments.

## AUTHOR CONTRIBUTIONS

**Wei Chen:** Conceptualization; investigation; writing – original draft; validation; visualization; writing – review and editing; formal analysis; project administration; data curation. **Jacob M. Shaffer:** Investigation; writing – review and editing; writing – original draft; visualization; formal analysis; data curation. **Christine D. Keating:** Funding acquisition; writing – review and editing; supervision; resources. **Scott A. Showalter:** Funding acquisition; writing – review and editing; supervision; resources; conceptualization.

## Supporting information


**Figure S1.** ^13^C, ^15^N‐CON spectra of CTD12 under different conditions overlaid with the no‐crowder reference spectrum (425 μM CTD12). Overlays are shown for (a) 1 mM CTD12 with no crowder, (b) 100 g/L PEG, (c) 100 g/L dextran, (d) 100 g/L BSA, (e) 100 g/L lysozyme, and (f) 100 g/L reconstituted *E. coli* cytosol.
**Figure S2.** Comparison of residue‐level effects of PEG and ethylene glycol (EG) monomer on CTD12. Overlay of ^1^H–^15^N HSQC spectra of CTD12 in the absence of crowder (black), 30% (w/v) PEG (purple), and 30% (w/v) EG (teal). Corresponding residue‐level chemical shift perturbation (CSP) profiles are shown for ^15^N and ^1^H. PEG and EG both induced substantial SCPs but with distinct residue‐level patterns. CTD12 concentrations were 1.7 mM (no crowder), 518 μM (30% PEG), and 300 μM (30% EG). Buffer conditions: 25 mM sodium phosphate (pH 6.2), 50 mM NaCl, 10% D_2_O, and 1 mM DSS.

## Data Availability

The data that support the findings of this study are available from the corresponding author upon reasonable request.
